# A Three-Layer Network Model of Direction Selective Circuits in the Optic Tectum

**DOI:** 10.3389/fncir.2017.00088

**Published:** 2017-11-21

**Authors:** Fatima Abbas, Marcus A. Triplett, Geoffrey J. Goodhill, Martin P. Meyer

**Affiliations:** ^1^Centre for Developmental Neurobiology and MRC Centre for Neurodevelopmental Disorders, Institute of Psychiatry, Psychology and Neuroscience, King's College London, London, United Kingdom; ^2^Queensland Brain Institute and School of Mathematics and Physics, University of Queensland, St Lucia, QLD, Australia

**Keywords:** direction selectivity, tectum, retinal ganglion cell, network model, zebrafish, functional imaging

## Abstract

The circuit mechanisms that give rise to direction selectivity in the retina have been studied extensively but how direction selectivity is established in retinorecipient areas of the brain is less well understood. Using functional imaging in larval zebrafish we examine how the direction of motion is encoded by populations of neurons at three layers of the optic tectum; retinal ganglion cell axons (RGCs), a layer of superficial inhibitory interneurons (SINs), and periventricular neurons (PVNs), which constitute the majority of neurons in the tectum. We show that the representation of motion direction is transformed at each layer. At the level of RGCs and SINs the direction of motion is encoded by three direction-selective (DS) subtypes tuned to upward, downward, and caudal-to-rostral motion. However, the tuning of SINs is significantly narrower and this leads to a conspicuous gap in the representation of motion in the rostral-to-caudal direction at the level of SINs. Consistent with previous findings we demonstrate that, at the level of PVNs the direction of motion is encoded by four DS cell types which include an additional DS PVN cell type tuned to rostral-to-caudal motion. Strikingly, the tuning profile of this emergent cell type overlaps with the gap in the representation of rostral-to-caudal motion at the level of SINs. Using our functional imaging data we constructed a simple computational model that demonstrates how the emergent population of PVNs is generated by the interactions of cells at each layer of the tectal network. The model predicts that PVNs tuned to rostral-to-caudal motion can be generated via convergence of DS RGCs tuned to upward and downward motion and feedforward tuned inhibition via SINs which suppresses responses to non-preferred directions. Thus, by reshaping directional tuning that is inherited from the retina inhibitory inputs from SINs can generate a novel subtype of DS PVN and in so doing enhance the encoding of directional stimuli.

## Introduction

The ability to detect motion, whether it results from movement of the observer or another object, is perhaps the most important task performed by the visual system. It is essential for catching prey, avoidance behaviors, and provides a rich source of information that is used for navigation. Many neurons at various stages of the visual pathway respond selectively to the direction of moving stimuli and in the vertebrate retina the circuit mechanisms that generate direction selectivity have been studied extensively (Borst and Euler, 2011; Vaney et al., 2012; Mauss et al., 2017). However, the mechanisms that generate direction selectivity in areas downstream of the retina are less well understood.

The optic tectum (OT) and its mammalian homolog, the superior colliculus (SC), are evolutionarily conserved retinorecipient structures involved in controlling gaze shifts and body movements relative to salient visual stimuli (Angeles Luque et al., 2005; Gahtan et al., 2005; Saitoh et al., 2007; Gandhi and Katnani, 2011; Bianco and Engert, 2015; Temizer et al., 2015; Dunn et al., 2016). Both the OT and SC receive topographically organized input from retinal ganglion cells (RGCs), including those that are direction selective (Maximov et al., 2005; May, 2006; Huberman et al., 2008; Kay et al., 2011; Gabriel et al., 2012; Nikolaou et al., 2012). Selectivity for motion direction has also been observed in postsynaptic neurons within the OT and SC and the mechanisms by which these neurons acquire their tuning have recently been investigated. In the mouse SC for example, direction selective neurons acquire their selectivity as a result of precisely converging excitatory inputs from similarly tuned RGCs. The direction selective retinal input is further amplified in SC neurons by intracollicular inputs without changing the preferred direction or degree of tuning (Shi et al., 2017). Further evidence of a retinal origin of direction selectivity comes from studies of the zebrafish tectum which show that the dendrites of direction selective periventricular neurons (PVNs), which constitute the majority of cells within the tectum, ramify within the tectal laminae that are targeted by directionally tuned RGC axons (Gabriel et al., 2012; Nikolaou and Meyer, 2015). Furthermore, the study by Gabriel et al. shows that the excitatory input and spike output of direction PVNs are matched (Gabriel et al., 2012). Together these observations suggest that, similar to the mouse SC, direction selectivity in the tectum is established by tuned excitatory input from the retina. Although, Gabriel et al. demonstrate that direction selective PVNs also receive inhibitory input tuned to the non-preferred direction it is likely that this serves to sharpen, rather than establish directional tuning in tectal neurons. However, a separate study using whole cell recordings found that tectal cells receive inhibitory inputs that are strongly biased toward the non-preferred direction of motion, whereas the excitatory inputs show little selectivity (Grama and Engert, 2012). This contrasts with the studies above by indicating that direction-selectivity is generated via tuned inhibition and that it emerges within the tectum. These contrasting findings can perhaps be reconciled by population functional imaging studies which show that cells tuned to motion in the upward, downward, and caudal-to-rostral directions are present in the population of RGCs that target the tectum and also PVNs, but that a fourth subtype of PVN tuned to motion in the rostral-to-caudal emerges within the tectum (Hunter et al., 2013). Thus, the tuning of three subtypes of direction selective PVNs is likely inherited from the retina while a fourth subtype is established *de novo* by intratectal mechanisms.

**Figure 1:**
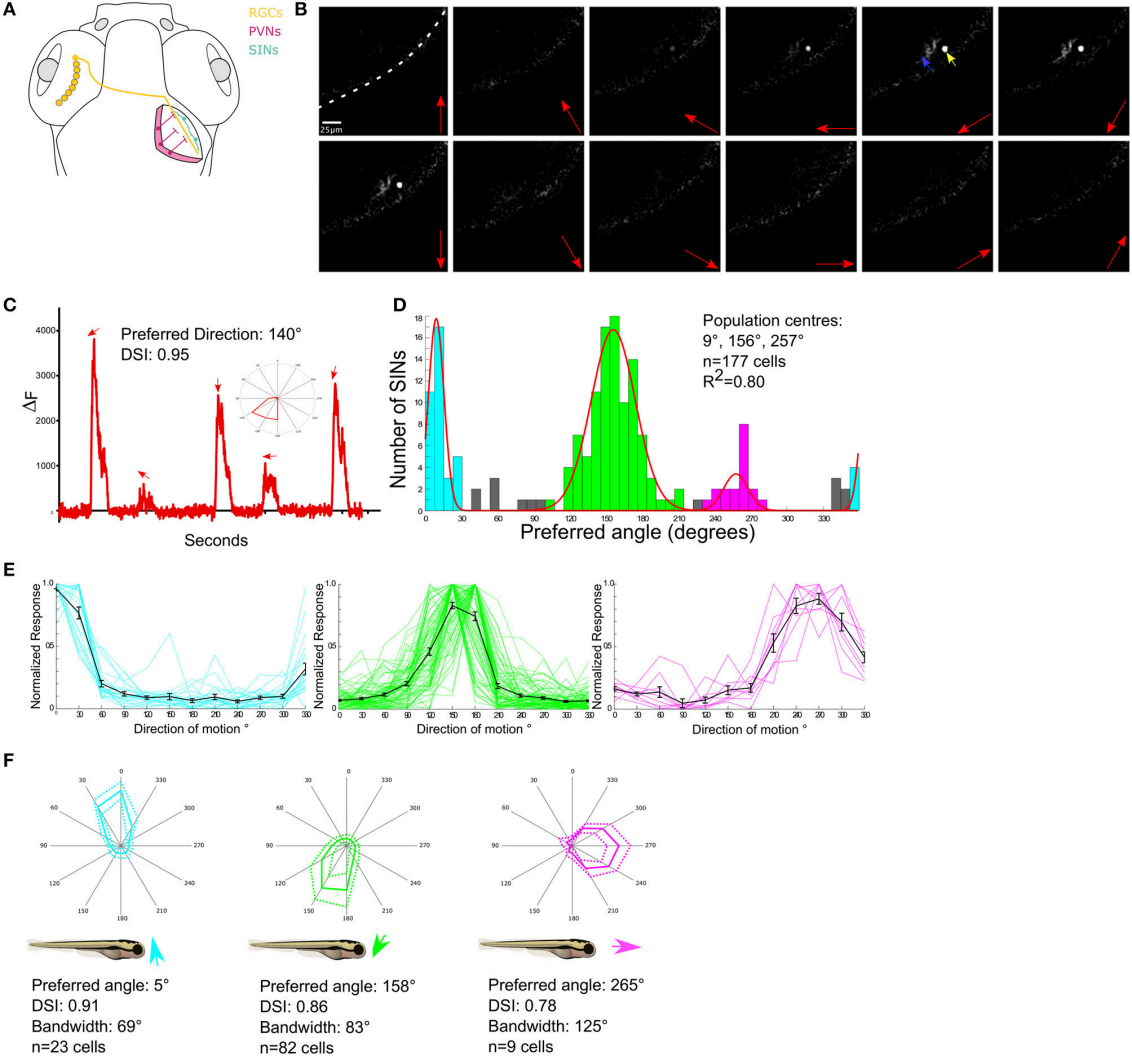
Functional characterization of DS SINs. **(A)** Diagram showing dorsal view of the retinotectal projection in zebrafish larvae. Retinal ganglion cells (in yellow) send projections contralaterally from the retina to the neuropil of the optic tectum where they arborize. Periventricular neurons (PVNs, in pink) project dendrites into the tectal neuropil. Unlike PVNs, Superficial inhibitory interneurons (SINs) (cyan) have cell bodies located in the most superficial tectal neuropil and extend broad monostratified arbors into the retinorecipient layers. **(B)** Responses of a single SIN expressing GCaMP5G to a drifting grating stimulus. Red arrows indicate direction of grating motion, yellow arrow indicates SIN cell body and blue arrow indicates SIN arbor. White dashed line indicates skin covering the tectum. **(C)** Example response of a single SIN tuned to 140° directed motion. Directions of motion eliciting significant responses are indicated by arrows. Inset shows response as a polar plot. **(D)** Cumulative histogram of preferred directions of all DS SINs. Fitting von-Mises curves (red lines, *R*^2^ = 0.8) to the population histogram reveals three normally distributed, non-overlapping populations with population peaks centered at 9°, 157°, and 264°. Each population is color-coded according to preferred direction. **(E)** Normalized responses of direction selective SINs, color-coded according to subtype. Responses of individual cells are shown as colored lines, mean responses shown in black. **(F)** Polar plots showing normalized responses of each population; mean (solid line) and dashed (±1 *SD*). Mean DSI, tuning bandwidth (full width half max) and preferred direction of each population relative to the body axis of the fish are shown below each polar plot.

Here we investigate potential circuit mechanisms that generate the emergent population of PVNs tuned to rostral-to-caudal motion. Using functional imaging and transgenic expression of the genetically-encoded reporter GCaMP5 we determine the directional tuning properties of neurons at three stages of the tectal network: RGC inputs to the tectum, a layer of superficial GABAergic interneurons (SINs), the cell bodies of which are located at the dorsal surface of the tectum, and PVNs which reside in deeper layers of the tectum (Figure 1A). We confirm the presence of three types of direction selective RGC, three types of direction selective PVN whose preferred directions match those of the RGC inputs, and also the emergent population of PVN tuned to rostral-to-caudal motion. We also describe three subtypes of direction selective SINs whose preferred directions match those of the RGCs but whose tuning is narrower. This narrowing reshapes the retinal representation of motion at the level of SINs leaving a gap in the representation of motion in the rostral-to-caudal direction. Using these new data we construct a three-layer computational model of the interactions between RGCs, SINs, and PVNs. In this model the PVNs tuned to upward, downward, and caudal-to-rostral motion inherit their tuning from similarly tuned RGCs. Furthermore, the model predicts that the PVNs tuned to rostral-to-caudal motion can be generated via convergence of direction selective RGCs tuned to upward and downward motion and feedforward tuned inhibition via SINs which suppresses responses to non-preferred directions.

## Materials and methods

### Zebrafish

All work was approved by the local Animal Care and Use Committee, King's College London, and was performed in accordance with the Animals (Experimental Procedures) Act, 1986, under license from the United Kingdom Home Office. Functional imaging experiments were carried out on zebrafish in the nacre mutant background. These larvae have reduced skin pigmentation due to a loss of neural crest derived melanophores (Lister et al., 1999), but retain the pigmented epithelium in retina which is necessary for normal vision. Larval zebrafish were kept at 28.5°C on a 14 h on/10 h off light cycle. To express GCaMP5 in SINs and RGCs the transgenic lines *Tg(s1156t:Gal4)* (Scott et al., 2007) and *Tg(Isl2b:Gal4)* (Ben Fredj et al., 2010), respectively were crossed with a *Tg(UAS:GCaMP5)* line. The transgenic line *Tg(elavl3:GCaMP5)* (Ahrens et al., 2013) was used to image visually evoked responses in tectal neurons.

### Functional imaging

All imaging was carried out using an LSM 710 confocal microscope equipped with a spectral detection scan head and a Plan-Apochromat 20x/1.0 NA water immersion objective (Carl Zeiss). GCaMP5 imaging was carried out using a 488 nm laser and with the pinhole aperture set to 1 Airy Unit. All data were acquired as 12-bit time series, recorded at ~4 Hz with image dimensions of 256 × 256 pixels. Pixel dimensions ranged from 0.42 μm^2^ for RGC imaging to 0.81 μm^2^ for imaging tectal neuron cell bodies. Larvae were mounted dorsal side up on a custom slide in 2% low-melting point agarose (Sigma, UK) made in Danieau solution. This was sufficient to immobilize the larvae whilst imaging without the need for anesthesia. The agarose was removed from in front of the right eye facing the projector screen to allow an unobstructed view. Larvae were placed in a custom-made imaging chamber with diffusive filter 3026 (Rosco Inc., Hollywood, CA) fitted to one side as a projection screen. This was filled with Danieau and the larva was placed on a platform within this chamber positioned at 30 mm from the screen.

### Visual stimuli

Visual stimuli were delivered via a DLP pico projector (Optoma). The projected image covered ~97° × 63° degrees of the visual field. Stimuli were displayed at a luminance of 25 and 175% (corresponding to 8–56 cd/m^2^ of the mean gray luminance of the background, 32 cd/m^2^). Stimuli were generated and controlled by custom written Matlab (Mathworks) and LabVIEW code and driven by ViSaGe (Cambridge Research Systems, UK) stimulus presenter. To determine direction selectivity sinusoidal gratings drifting in 12 different directions were presented in a pseudo random order. The direction of motion was orthogonal to the long axis of the gratings. The sinusoidal gratings had a fade in and out time of 3 s to ensure that fluorescence signal from previous epochs had decayed before the next epoch is presented. A spatial frequency of 0.05 cycles/° was used, with a temporal frequency of 1 cycle/second for all experiments-stimulus parameters known from previous experiments to elicit reliable responses from neurons in the tectum (Nikolaou et al., 2012; Hunter et al., 2013). A randomly placed blank epoch lasting 2 s, in which a mean gray background is displayed was also included to aid in baseline fluorescence calculation.

### Imaging processing and analysis

All time series experiments were first corrected by realigning images using a rigid body algorithm (spm8, http://www.fil.ion.ucl.ac.uk/spm/) to remove artifacts from movement of the larvae or drift during recording. Median filtering with a kernel size of one voxel was used to remove dark and shot noise. Spatial smoothing with a 2D Gaussian kernel of two voxels was used to improve signal to noise. Low frequency baseline drifts in fluorescence were corrected using a cubic spline algorithm extrapolating between knots averaged from 5 s of interepoch-interval data. Signal intensity changes were calculated at each voxel, and integral responses during the epochs calculated to produce a response value for each experiment epoch. Direction and orientation selective indices (DSI and OSI) were calculated based on fitted von-Mises profiles. DSI and OSI are calculated using responses at preferred angle (R_pref_), null direction (R_null_), preferred orientation (R_prefori_), and the orthogonal to this orientation (90° from preferred orientation, R_orth_). DSI = (R_pref_ − R_null_)/(R_pref_ + R_null_), OSI = (R_prefori_ − R_orth_)/(R_prefori_ + R_orth_). An estimate of goodness of fit of the von-Mises curve, *R*^2^, was also calculated. Direction selective responses were determined based on stringent criteria; Direction selective: DSI > 0.5, OSI < 0.5, R2 > 0.7; Orientation selective: DSI < 0.5, OSI > 0.5, R2 > 0.7. The preferred direction of motion was obtained from the center of the fitted curve. To derive the number of subtypes of direction RGC, SIN and PVN average direction selective fitted angles were calculated for each cell/voxel soma and plotted on a cumulative histogram summarizing the incidence over binned preferred angles (0–360°). Multiple von-Mises curves were fitted to cumulative histograms using a multidimensional constrained nonlinear minimization approach, with peak-center, height, concentration as free dimensions. The bandwidth of the curves was used to define the preferred angle bounds between which the cells within these populations lie. Polar plots were generated of the mean responses of cells within each of these populations to all 12 angles presented during experiments, with S.D.s also plotted. The bandwidth of each cell's responses was also calculated as the full width at the half maximum of the cell's responses.

### Modeling

The network model consisted of 10 rate-based units with rectified-linear activation functions. We denote the firing rates of the RGC units by *r*_*i*_, the SIN units by *s*_*i*_, and the PVN units by *p*_*i*_. The rate dynamics followed the differential equations

(1) $$\tau \frac{dr_{i}}{dt}=\;-r_{i}+f\left(\theta ;\;\theta_{i},\kappa_{i}\right)$$

(2) $$\tau \frac{ds_{i}}{dt}=\;-s_{i}+\sigma \left(\sum_{j}w_{ij}^{sr}r_{j}-\;\sum_{j}w_{ij}^{ss}s_{j}\right)$$

(3) $$\tau \frac{dp_{i}}{dt}=\;-p_{i}+\sigma \left(\sum_{j}w_{ij}^{pr}r_{j}-\;\sum_{j}w_{ij}^{ps}s_{j}\right)$$

where θ_*i*_ is the preferred stimulus direction for RGC i, σ(*x*) = max(*x*, 0) is a linear rectifier, and *f*(θ; θ_*i*_, κ_*i*_) is the von Mises distribution with mean θ_*i*_ and concentration κ_*i*_. The parameters for the von Mises distribution were obtained using the CircStat MATLAB toolbox as the estimated maximum likelihood fit to the RGC population tuning data, with subsequent adjustment of the concentration parameters to align the resulting preferred stimulus direction of P2 with the experimentally identified direction. We assigned each element of the weight matrix *w*^*xy*^ to 1 whenever we asserted a corresponding connection existed in the circuit schematic, and then divisively normalized the matrix so that its entries summed to 1. This caused all connections leaving a given cell-type to have the same strength. Polar plots of tuning curves were obtained by computing a unit's stabilized response to each input direction, and then normalizing the resulting vector of responses to take values between 0 and 1. Response amplitude, DSI, FWHM bandwidth, and tuning error were calculated from the unnormalized tuning data.

## Results

### Analysis of directional tuning in SINs, RGCs, and PVNs

Directional tuning in RGCs, SINs, and PVNs have been described previously (Nikolaou et al., 2012; Hunter et al., 2013). However, these earlier studies used different probes to study each population; RGCs targeting the tectum were studied using SyGCaMP3- a genetically-encoded, presynaptically localized form of GCaMP3, while SINs and PVNs were characterized by bulk injection of the calcium sensitive-dye, Oregon Green 488 BAPTA-1AM. To ensure that any differences in tuning properties reflect real cell-type differences rather than differences in the properties of the probes or the method of their introduction, we re-examined direction selectivity by expressing the genetically encoded calcium sensor, GCaMP5G, in each population.

#### SINs

To drive expression of GCaMP5G in SINs the transgenic lines, *Tg (s1156t:Gal4)* (Scott et al., 2007) and *Tg(UAS: GCaMP5)* were crossed. This resulted in a mean of 26 SINs per tectal hemisphere which represents ~10% of all the cell bodies located within the neuropil (data not shown). The labeling was sufficiently sparse to resolve single SIN cell bodies (Figure 1B). To examine the tuning properties of individual SINs drifting sinusoidal gratings moving in each of 12 different directions were presented to one eye while visually evoked responses were imaged in the contralateral tectum (Figure 1B). To characterize directional tuning the integral response during each stimulus epoch was calculated and this was then used to generate tuning curves for each cell (Figure 1C). The center of the fitted curve used to estimate DSI was also used to provide an estimate of the preferred direction of motion. Of 190 visually responsive SINs 177 were found to be direction selective (hereafter referred to as DS), eight were selective for grating orientation (OS) (data not shown) and five were un-tuned. The preferred angles of DS SINs were used to generate a population histogram (Figure 1D). These revealed distinct distributions of preferred angles. By iteratively fitting three summed von-Mises distributions to the population histograms (overlaid color curves in Figure 1D), we found that there are three, non-overlapping, DS subpopulations with peaks centered at 9° (upward motion), 156° (downward motion), and a smaller population centered at 257° (caudal-to-rostral). SINs selective for upward and downward motion have been identified previously using Oregon Green 488 BAPTA-1AM, but the minor population tuned to caudal-to-rostral motion has not previously been described (Hunter et al., 2013). The population histogram was used to define subtypes of DS SIN (see section Methods) and population tuning curves (Figure 1E) and polar plots illustrating the mean (±1 *SD*) normalized response profile for each DS subtype (Figure 1F) were generated. These allowed us to summarize the population mean tuning properties (preferred angle, direction-selectivity index, and tuning bandwidth) for each subtype of DS SIN (Figure 1F).

#### RGCs

**Figure 2:**
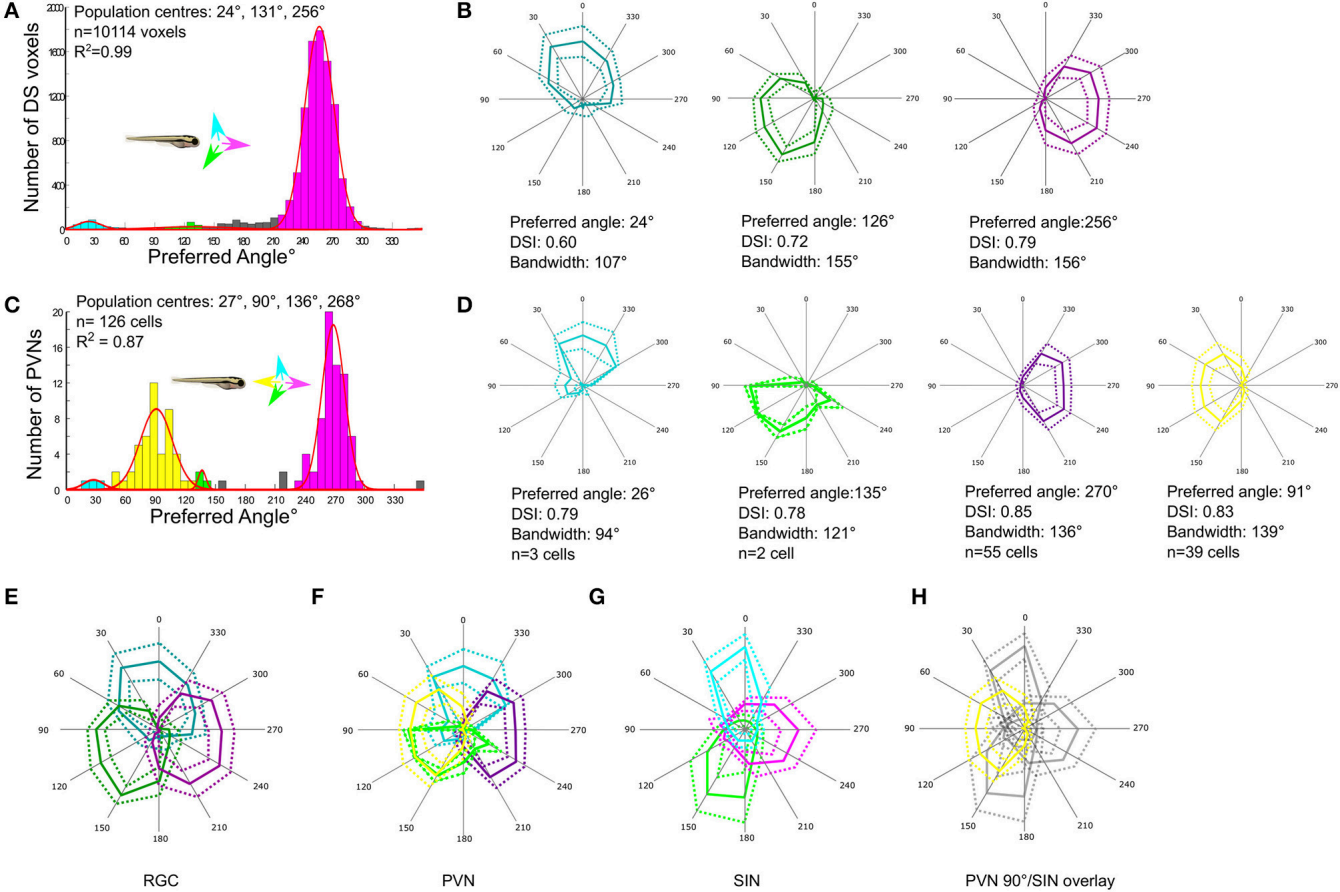
Comparison of direction-selectivity in the SIN, RGC and periventricular neuron (PVN) populations. **(A)** Cumulative histogram of preferred directions of all RGC voxels (*n* = 10,114 voxels from 29 larvae). Fitting von-Mises curves (red lines, *R*^2^ = 0.99) to the population histogram reveals three normally-distributed, non-overlapping populations with population peaks centered at of 24°, 131°, and 256°. Each population is color-coded and the preferred direction of motion of each population relative to the body axis of the fish is shown in inset. **(B)** Polar plots showing normalized responses of each DS RGC subtype; mean (solid line) and dashed (±1 *SD*). Mean DSI, tuning bandwidth (full width half max) and preferred direction of each DS RGC subtype are shown below each polar plot. **(C)** Histogram of DS PVN preferred directions (*n* = 126 tectal neurons from 24 fish). Four normally distributed populations are present, with populations centered at 27°, 90°, 136°, and 268° (*R*^2^ = 0.87). Note the emergent population of PVN tuned to 90° motion (yellow) that is absent from both RGCs and SINs. **(D)** Polar plots showing normalized responses of each DS PVN subtype; mean (solid line) and dashed (±1 *SD*). Mean DSI, tuning bandwidth (full width half max) and preferred direction of each subtype are shown below each polar plot. **(E–H)** Overlaid polar plots summarizing how direction of motion is encoded by RGCs **(E)**, PVNs **(F)**, and SINs **(G)**. RGCs exhibit a tiled and triangular representation of directional space by three subtypes of DS RGC. In the SIN population the representation of motion is reshaped, leaving a gap at 90°. Directional space is represented by 4 subtypes of PVNs with preferred directions aligned with the cardinal axes. **(H)** Overlaying the polar plots of DS SINs with the emergent population of PVN reveals that the gap in the representation of motion in the rostral-to-caudal direction in the SIN population aligns with the emergent population of PVNs tuned to 90°.

To functionally characterize RGCs targeting the tectum GCaMP5 was expressed in RGCs specifically by crossing *Tg(Isl2b:Gal4)* with *Tg(UAS:GCaMP5)*. To characterize directional tuning in RGC axons, we used the imaging and analysis strategies described above (and see section Methods). The histogram of the preferred angles of all DS RGC voxels reveals three DS RGC subpopulations with minor peaks centered at 24° (upward motion) and 131° (downward motion), and the largest population centered at 256° (caudal-to-rostral) (Figure 2A). By generating polar plots illustrating the mean (±1 *SD*) normalized response profile for each DS RGC subtype (Figure 2B) we find that the population mean tuning properties (preferred angle, direction-selectivity index, and tuning bandwidth) for each DS RGC subtype are very similar to those described previously (Nikolaou et al., 2012).

#### PVNs

Directional tuning within the population of PVNs within the tectum expressing GCaMP5G using *Tg(elavl3:GCaMP5G*) was analyzed using the stimulus set and analysis protocol described above. This reveals four subtypes of DS PVN tuned to upward, downward, and caudal-to-rostral motion and also the emergent population tuned to rostral-to-caudal (Figure 2C). The tuning properties of each DS PVN subtype also closely match those described previously (Hunter et al., 2013; Figure 2D).

### Transformation of encoding at three layers of the tectal network

Having determined the tuning profiles of DS RGCs, SINs, and PVNs we compared how the direction of motion is encoded at these three levels of the tectal network. At the level of RGCs, the direction of motion is encoded by three subtypes of RGC with preferred directions separated by ~120° and which together tile direction space (Figure 2E). At the level of tectal PVNs this representation is transformed into an overlapping cardinal representation by four subtypes of tectal neuron, with an emergent population tuned to rostral-to-caudal (90°) motion (Figure 2F). However, the tuning properties of SINs described here differ slightly from those previously described (Hunter et al., 2013). In agreement with Hunter et al. (2013) we demonstrate the presence of two DS subtypes tuned to upward and downward motion; however, we also find a population tuned to forward motion that has not been described (Figure 2G). Furthermore, we also find that the tuning bandwidths of DS SINs, particularly those tuned to upward and downward motion are narrower than that seen in the DS RGCs (upward selective: RGC 107°, SIN 69°; downward selective: RGC 155°, SIN 83°; caudal-to-rostral selective: RGC 156°, SIN 125°) and this narrowing generates a gap in the representation of motion in the rostral-to-caudal direction at the level of SINs. Overlaying the polar plots of DS SINs with the emergent population of PVNs reveals that the gap in the representation of motion in the rostral-to-caudal direction at the level of SINs aligns with the preferred angle of PVNs tuned to this direction (Figure 2H).

### Modeling the interactions between RGCs, SINs, and PVNs

**Figure 3:**
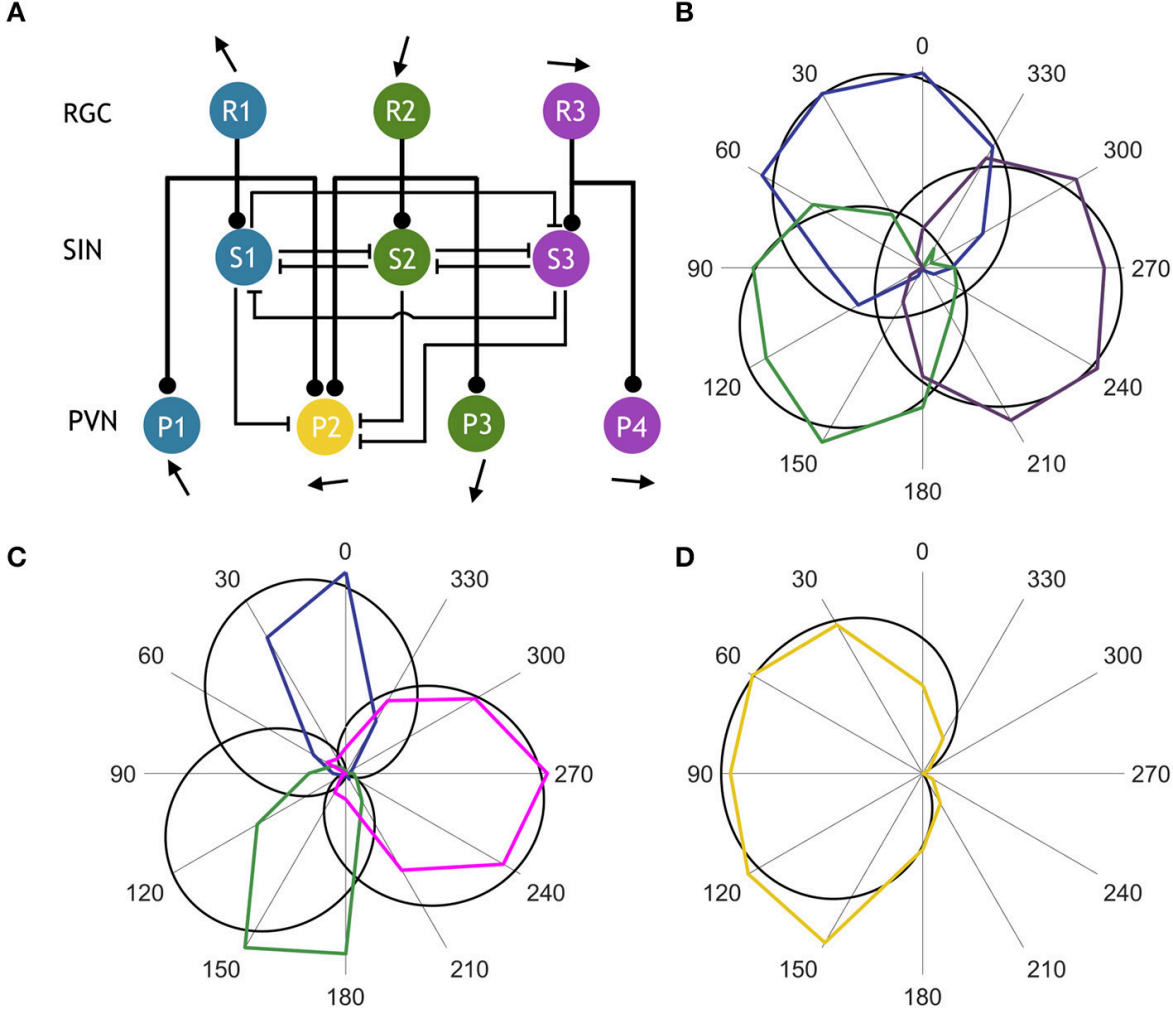
Computational model of the direction selective tectal circuit. **(A)** Circuit motif showing a population of PVNs integrating input from two RGC populations and three SIN populations. Rounded arrowheads represent excitatory connections, flat arrowheads represent inhibitory connections, and pointed arrows indicate preferred stimulus direction. **(B)** Comparison between experimentally observed (colored) and simulated (black) normalized RGC tuning curves. **(C)** Normalized tuning curves of observed (colored) vs. simulated (black) SINs. **(D)** Normalized tuning curves of observed (colored) vs. simulated (black) PVN population tuned to rostral-to-caudal directed motion.

**Figure 4:**
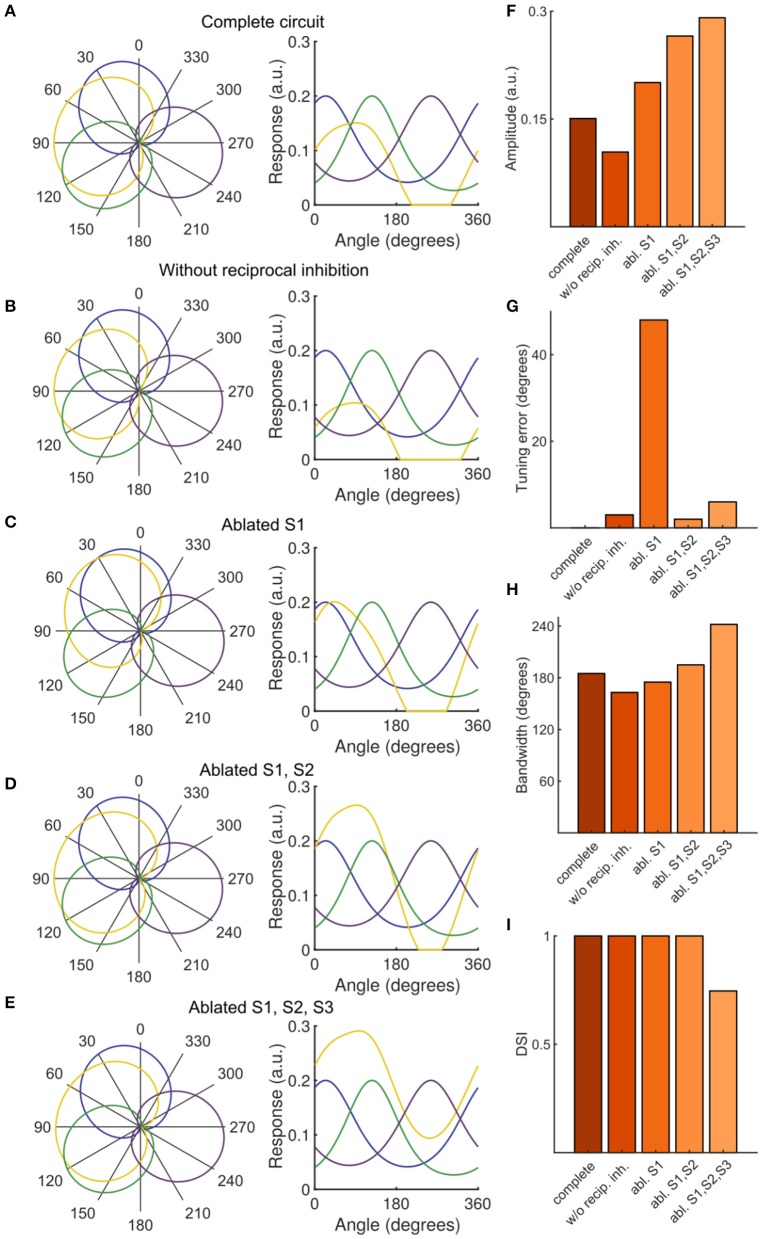
Contribution of SINs to stimulus tuning in simulated PVN populations. **(A–E)** PVN population tuning curves under various circuit perturbations, presented as polar plots with normalized data (left) and plots with unnormalized data (right). **(F)** Amplitude of PVN type P2 (the emergent population tuned to rostral-to-caudal directed motion) under simulated manipulations. **(G)** Tuning error of P2, defined as the absolute difference between the experimentally observed direction preference and the simulated direction preference. **(H)** FWHM bandwidth of P2 tuning curve. **(I)** DSI of P2.

Using our functional imaging data we constructed a simple computational model of the direction selective tectal circuit that demonstrates how the preference for direction of motion in the rostral-to-caudal direction could emerge from the interaction between different cell types. The model consisted of a three-layer network, with an RGC layer providing feedforward excitation to a PVN layer and an intermediate SIN layer (Figure 3A). As in other recent work (Naumann et al., 2016), each unit in the model represents one functional population of neurons, rather than an individual neuron (see Discussion). The SIN populations reciprocally inhibited each other which narrowed the tuning curves of the SINs relative to the RGCs (Figures 3B,C). The SINs also inhibited responses to caudal-to-rostral, upward, and downward directed motion (but not rostral-to-caudal motion- see gap in representation of backward motion by SINs, Figure 2G) within the population of PVNs tuned to rostral-to-caudal motion (labeled P2). The RGCs were assigned von Mises tuning curves with means of 24°, 124°, and 256°, matching the preferred stimulus directions identified in Figure 2B. Population P2 received input from the two RGC populations tuned to upward and downward motion (R1 and R2) centered at 24° and 124°, while the other PVNs received input from unique populations of DS RGCs and thereby inherited their stimulus tuning directly. By integrating the excitatory input from two retinal pathways, the PVN population P2 developed a preferred stimulus direction of 91° (Figure 3D), recreating the emergent preference of rostral-to-caudal motion reported here and previously (Hunter et al., 2013). The SINs, tuned to the three remaining cardinal directions, sharpened tuning of P2 by suppressing responses to the non-preferred stimulus directions (Figure 3D). To further probe the role of the SINs in forming the novel direction preference, we perturbed the intact circuit (Figure 4A) by simulating a series of manipulations. We first eliminated the reciprocal SIN connections and found that this caused a reduction in the response amplitude and bandwidth of P2 due to increased levels of feedforward inhibition (Figures 4B,F,H). We then systematically ablated the SIN populations and measured the effect that this had on the tuning properties of P2. Successive manipulations generally increased both the response amplitude (Figure 4H) and the tuning bandwidth (Figure 4H) of P2. Ablation of S1 (Figure 4C) resulted in a misalignment between excitation and inhibition in the synaptic input to P2, causing a 45° shift in the preferred stimulus direction of P2 relative to the complete circuit (Figure 4G). Further eliminating S2 (Figure 4D) rebalanced the excitatory input to P2 and increased its bandwidth and maximum response amplitude (Figures 4F–H). Simultaneously ablating S1, S2, and S3 (Figure 4E) relieved the circuit of all inhibition, yielding the greatest increase in bandwidth and amplitude, but with a significant degradation in direction-selectivity (Figures 4F,G,I). These manipulations show that deviations from the complete circuit with small errors in tuning either reduce the P2 PVN population response amplitude or increase the FWHM bandwidth of its tuning curve, suggesting that while SIN inhibition may not be essential for selectivity to motion detection in the rostral-to-caudal direction it is essential for refining stimulus encoding.

## Discussion

Here we explore a potential circuit mechanism for generating an emergent population of DS neuron in the optic tectum of larval zebrafish. Using population functional imaging we confirm previous findings by showing that at the level of RGCs the direction of motion is encoded by three DS subtypes with preferred angles separated by ~120° (a triangular representation) and that at the level of PVNs the retinal representation is transformed into a cardinal representation (four DS PVN subtypes with preferred angles separated by ~90°; Nikolaou et al., 2012; Hunter et al., 2013). However, the tuning properties of SINs described here differ in two respects from our previous description. Firstly we identify a novel DS SIN subtype tuned to caudal-to-rostral motion and secondly we find that the tuning profiles of SINs appear to be narrower than that described in earlier work. This narrowing of tuning leads to a gap in the representation of motion in the rostral-to-caudal direction at the level of the SIN population. A possible explanation for the different findings in this and our previous study is that different probes were used. In our previous study SINs were characterized using bolus injections of the calcium sensitive dye, Oregon Green 488 BAPTA-1, AM (OGB), and SIN soma were segmented by spatially aggregating DS voxels into cell body-sized islands of like responses. Because OGB injections also label the neuropil surrounding SIN neurons it may be that signal originating from the dendrites of DS PVNs and RGC axons (which also terminate in the superficial retinorecipient layers) were incorporated into the functionally defined SINs. This may have resulted in an apparently larger tuning bandwidth for DS SINs. Furthermore, SINs tuned to caudal-to-rostral may have been missed in our previous study because their soma may have been hard to distinguish from the DS responses in the surrounding neuropil. The mosaic expression of GCaMP5G used in this study enables unambiguous recording from single SINs without contaminating signal from other labeled neurons.

Using our functional imaging data we modeled the interactions between the three layers of the tectal network. In the model SINs tuned to upward, downward and caudal-to-rostral motion inherit their tuning from the similarly tuned subtypes of DS RGCs. Reciprocal inhibition between the DS SINs narrows their tuning relative to the DS RGCs. Other mechanisms such as non-linearities in the spike generating mechanism, which we have not considered, may also contribute to the narrowing of SIN tuning. Our model predicts that a cell type whose tuning profile matches that of the emergent population of PVN type tuned to rostral-to-caudal motion can be generated by convergence of RGCs tuned to upward and downward motion. DS input from RGCs furthermore inhibits responses in the non-preferred directions via feedforward inhibition from SINs. Thus, in our model the tuning properties of the emergent population of PVN are established by DS excitatory input and inhibitory inputs that ultimately have a retinal origin. Our model is broadly consistent with the observations of Gabriel et al. who showed that DS PVNs receive DS excitatory and inhibitory inputs tuned to non-preferred directions (Gabriel et al., 2012). We extend this idea by suggesting that by sharpening directional tuning that is inherited from the retina inhibitory inputs from SINs can generate a novel subtype of DS PVN and in so doing enhance the encoding of directional stimuli in the tectum.

Our data also reveal striking changes in the relative sizes of DS populations at each layer of the tectal network. For instance, when compared to the RGC population there is a relative increase in SINs tuned to upward and downward motion and a relative decrease in cells tuned to caudal-to-rostral motion. This may be due to the fact that, compared to RGCs tuned to upward and downward motion, only a small fraction of RGCs tuned to caudal-to-rostral motion connect to SINs. Such subtype-specific differences in connectivity could account for the differences in relative sizes of each population at all layers of the tectal network. The differences in population sizes are not considered in our model in which each unit represents an entire population of neurons. One could imagine scaling the output of each unit by the size of the population it represents. However, this could be easily compensated for by proportionally scaling the synaptic weights of the connections from that unit onto other units so that it has the same net effect. Alternatively, one could simulate multiple units in each population, but again differences in population size could be normalized by scaling of the outgoing weights. Since we have no experimental data to directly constrain the relative sizes of the weights from each population in the model we argue that no new insight into the underlying mechanisms by which tuning curves are constructed would be gained by considering scaled population sizes.

Our model does not rule out alternative mechanisms for generating the emergent population of PVNs. For instance, the emergent population of PVNs may also be generated by combining inhibitory input from all DS SINs with un-tuned excitatory input (Grama and Engert, 2012). Tuned inhibition may also originate from other subtypes of PVNs rather than SINs. Indeed, a morphologically defined subtype of inhibitory DS PVN tuned to caudal-to-rostral motion has been described previously (Gabriel et al., 2012). Our model also makes predictions about how tuning bandwidth, preferred direction and response amplitude in PVNs tuned to rostral-to-caudal are altered when populations of DS SIN subtypes are ablated singly and in combination. These perturbations predict that, while SINs may not be essential for establishing direction selectivity in the emergent population of PVNs, they are essential for sharpening their tuning profile. The mechanisms and predictions of our model require testing experimentally, but this is currently very difficult to do since the genetic tools for selective targeting of SIN subtypes are currently not available (the s1156tGal4 line used in this study labels only a small fraction of SINs and those that are labeled are functionally diverse including SINs that exhibit size (Del Bene et al., 2010; Preuss et al., 2014) and orientation-selective tuning). However, as such tools become available our model will provide a useful framework for testing the diversity of mechanisms that generate direction selectivity in the tectum.

## Author contributions

FA and MM designed study. FA performed experiments and analyzed data. MT generated the model and FA, MT, GG, and MM wrote the paper.

### Conflict of interest statement

The authors declare that the research was conducted in the absence of any commercial or financial relationships that could be construed as a potential conflict of interest.
